# Synergistic co-delivery of diacid metabolite of norcantharidin and ABT-737 based on folate-modified lipid bilayer-coated mesoporous silica nanoparticle against hepatic carcinoma

**DOI:** 10.1186/s12951-020-00677-4

**Published:** 2020-08-18

**Authors:** Minchen Liu, Jue Tu, Yi Feng, Jiquan Zhang, Jing Wu

**Affiliations:** 1 Engineering Research Center of Modern Preparation Technology of TCM, Innovation Research Institute of Traditional Chinese Medicine, Shanghai University of Traditional Chinese Medicine, Shanghai, P. R. China; 2 Zhejiang Pharmaceutical College, Ningbo, P. R. China; 3 Academy of Chinese Medical Sciences, Zhejiang Chinese Medical University, Hangzhou, P. R. China; 4 Institute of Comparative Medicine, Zhejiang Chinese Medical University, Hangzhou, P. R. China

**Keywords:** Diacid metabolite of norcantharidin, ABT-737, Mesoporous silica nanoparticle, Lipid, Hepatic cancer

## Abstract

Diacid metabolite as the stable form of norcantharidin (DM-NCTD) derived from Chinese blister beetle (*Mylabris *spp.). The previous studies reported that DM-NCTD could enhance ABT-737-triggered cell viability inhibition and apoptosis in hepatocellular carcinoma (HCC) cell lines. To translate this synergistic therapy into in vivo anticancer treatment, a folate receptor-targeted lipid bilayer-supported chlorodimethyloctadecylsilane-modified mesoporous silica nanoparticle (FA-LB-CHMSN) with DM-NCTD loaded in CHMSN and ABT-737 in lipid bilayer was prepared, which could promote the cancer cell uptake of the drugs through folate receptor-mediated endocytosis. The structure and the properties of the nanoparticle were evaluated. FA-LB-CHMSN with DM-NCTD/ABT-737 loaded induced apparent tumor cell apoptosis and showed remarkably tumor inhibition in H22 tumor-bearing mice model, with significant cellular apoptosis in the tumor and no obvious toxicity to the tissues. We expect that this nanoparticle could be of interest in both biomaterial investigations for HCC treatment and the combination of chemotherapeutic drugs for synergistic therapies.

## Introduction

Hepatocellular carcinoma (HCC) can lead to cause of cancer-related death worldwide, as a most common cancer [1]. A few patients can be treated with surgery [2]. The palliative treatment is systemic chemotherapy, but the inefficiency in tumor-targeting and undesired effects limit its application [3, 4]. Small-molecule drug combination therapies are an attractive approach to enhancing cancer chemotherapeutic responses. The previous studies reported that Mcl-1 inhibitor diacid metabolite of norcantharidin (DM-NCTD), which derived from Chinese blister beetle (*Mylabris spp.*), could remarkably synergize the apoptotic response to BH3 mimetic agent ABT-737 in HCC cells with the influence of mitochondrial function [5, 6]. However, the application of DM-NCTD was limited by its short half-life, while ABT-737 was hampered by its poor physiochemical and pharmaceutical properties [7, 8]. Moreover, it is an obstacle for co-chemotherapeutic agents to be transferred to the same tumor cells [9].

Nanoparticles could carry multiple drugs into one formulation and co-delivery to the targeting site [10]. Herein, a folate acid (FA)-lipid bilayer (LB)–chlorodimethyloctadecylsilane (CH)-coated mesoporous silica nanoparticle (MSN) (FA-LB-CHMSN) was prepared, which could promote the cancer cell uptake of the drugs through folate receptor (FR)-mediated endocytosis [11, 12]. DM-NCTD was interacted with the positively charged amino-functionalized MSN through electrostatic forces, while ABT-737 was loaded in liposomal shell delivery. Through the construction of the nanoparticle, the two drugs can be delivered to tumor cells together for the first time. The structures and the properties of the nanoparticles were evaluated. Moreover, H22 cells treated with FA-LB(ABT-737)-(DM-NCTD@CHMSN) was drastically inhibited and apoptosis, while the ratio of JC-1 monomers was significant increased. We also demonstrated that FA-LB(ABT-737)-(DM-NCTD@CHMSN) provides safe and effective inhibition in a H22 tumor-bearing mice model.

## Method

Amino-functionalized MSN and be modified with CH were prepared as the methods reported [13, 14]. And then, CHMSN was loaded with DM-NCTD at 2.5:1 weight ratio. LB(ABT-737)-(DM-NCTD@CHMSN) and FA-LB(ABT-737)-(DM-NCTD@CHMSN) were prepared by modified thin film hydration method [15]. For the best therapeutic synergistic effect, ABT-737 was selected at 1:10 mol ratio to DM-NCTD [5, 6]. ABT-737 was added to the lipid mixture before the thin-film was hydrated [12]. The properties of nanoparticles were characterized. Cell viability, apoptosis and the mitochondrial activity of H22 cells were detected for the evaluation of in vitro antineoplastic activity. Cellular uptake study of H22 and AML12 cells were detected by using flow cytometry. Moreover, in vivo antitumor activity and preliminary toxicity were studied on the H22 tumor-bearing mice model [16–18]. The animal experiments were approved by the Animal Ethics Committee of Zhejiang Chinese Medical University. A detailed description of the methods and experiments is included in the Additional file: 1.

## Results and discussion

**Fig. 1:**
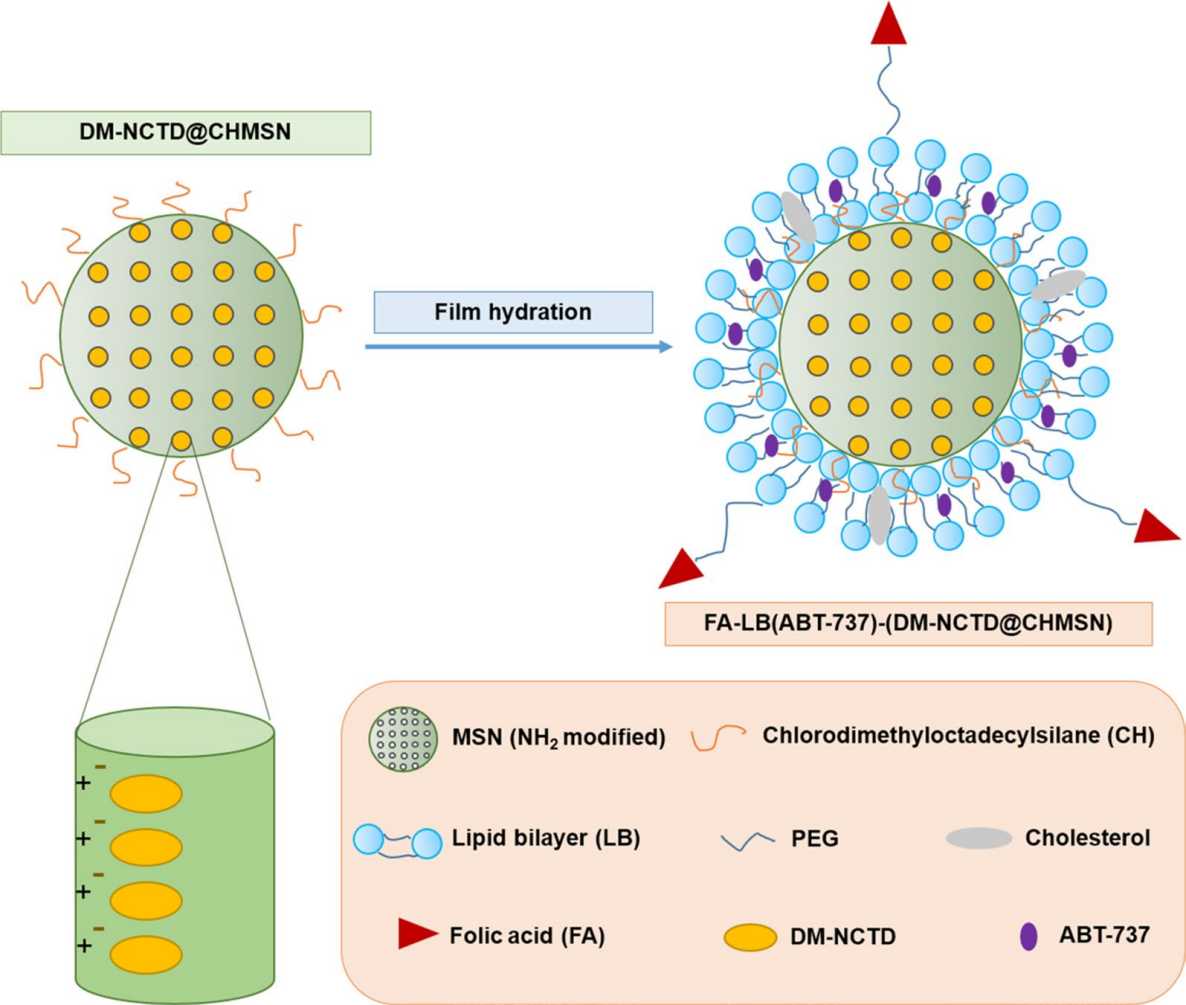
Schematic illustration of the synergistic co-delivery of diacid metabolite of norcantharidin and ABT-737 based on folate-modified lipid bilayer-coated mesoporous silica nanoparticle

**Fig. 2:**
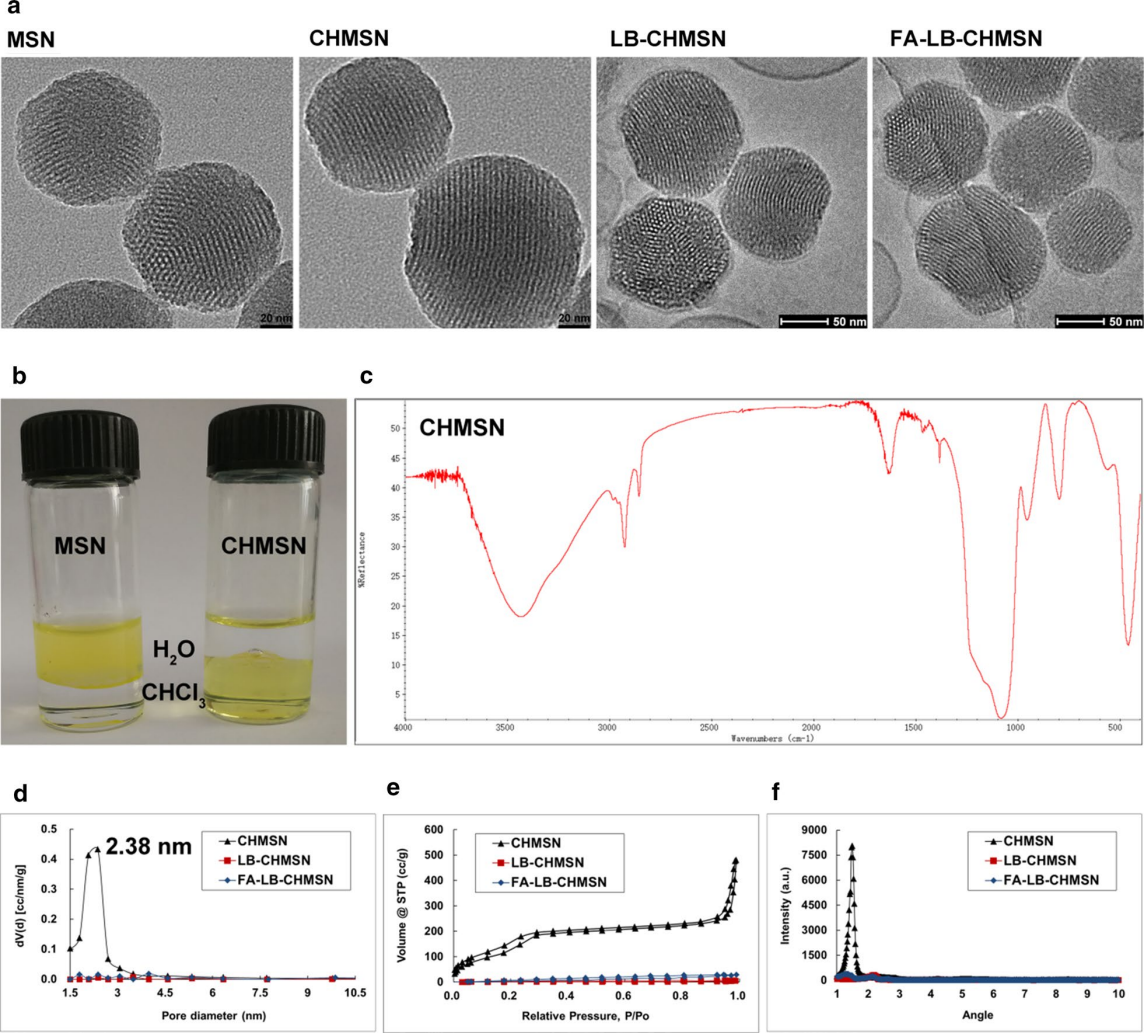
Characterization of the nanoparticles. **a** TEM of MSN and CHMSN; Cryo-TEM of LB-CHMSN and FA-LB-CHMSN. **b** Photograph of MSN and CHMSN suspended in water and CHCl_3_ mixed solvent. **c** FTIR spectra of CHMSN. **d** Pore size distribution, **e** N_2_ adsorption–desorption isotherm and **f** XRD patterns of CHMSN, LB-CHMSN and FA-LB-CHMSN

The application of MSNs is a recent development in biomaterial, due to its properties including excellent biocompatibility and easy surface modifications [19]. Although the strategies based on surface functionalization have been developed to improve the dispersibility and biocompatibility of silica nanoparticles, the functional groups still have the disadvantage of poor stability against aggregation in vivo. [20] The application of liposome could improve drug safety and enhance therapeutic efficacy, due to its biomimetic membrane and the ease in functionalization [21]. Furthermore, the surface modification with CH could enhance the stability of the core–shell structure in aqueous solutions and within the circulation in vivo. [19] FA specifically promotes cancer-cell uptake through FR-mediated endocytosis [11]. In this study, we employed FA-LB-CHMSN for co-loading with DM-NCTD and ABT-737 (Fig. 1). Transmission electron microscopy (TEM) images of MSNs and CHMSN and Cryo-TEM images of LB-CHMSN and FA-LB-CHMSN are shown in Fig. 2a. MSNs have a uniform spherical shape with an average diameter of about 120 nm, which porous structure contains a series of parallel channels can be clearly observed. Compared to CHMSN, it could be found that the modification of CH groups did not change the morphology of the MSNs. Besides, FITC was applied to observe the influence of CH before and after CHMSN was prepared. In Fig. 2b, it showed that CH-(FITC)@MSN could be dispersed in CHCl_3_, whereas the FITC@MSN could be suspended in H_2_O. From Fig. 2c, the successful modification of CH was shown by a C-H stretching absorbance at 2800–3000 cm^−1^ of CHMSN. Furthermore, LB on CHMSN was demonstrated by Cryo-TEM of LB-CHMSN and FA-LB-CHMSN with an intact liposomal shell of about 2 nm (Fig. 2a) and its particle size did not change in comparison with CHMSN, which were preferable for tumor accumulation due to the EPR effect [22]. Zeta potential of CHMSN was 31.8 mV, while zeta potential of LB-CHMSN or FA-LB-CHMSN was a neutral charge, which demonstrated that LB created a stable, protective circumstance for CHMSN. N_2_ adsorption–desorption and XRD analyses were performed to further understand the structure of those nanomaterials. In Fig. 2d–f, the N_2_ adsorption–desorption patterns of CHMSN with a narrow pore size of 2.38 nm. The surface area and pore volume of CHMSN were 980.17 m^2^/g and 0.9 cm^3^/g, respectively.

**Fig. 3:**
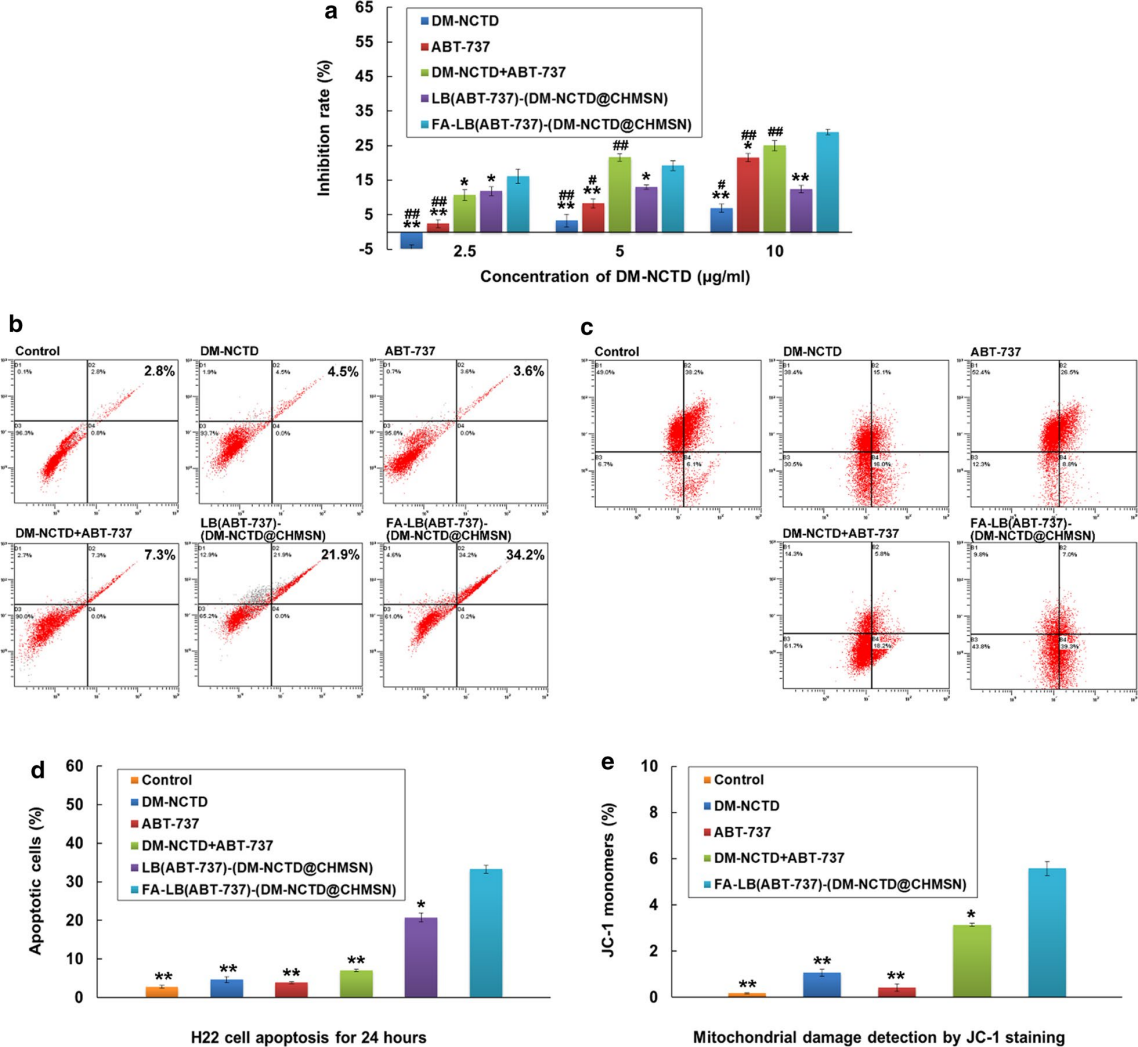
Evaluation of in vitro antineoplastic activity. **a** In vitro cytotoxicity of free drugs and nanoparticle samples on H22 cell lines for 24 h (n = 3). **b**, **d** Apoptotic effects of free drugs and nanoparticle samples in H22 cell lines (n = 3). **c**, **e** The mitochondrial activity was examined by JC-1 detection (n = 3). Notes: In vitro cytotoxicity study, the concentrations of DM-NCTD of DM-NCTD or DM-NCTD + ABT-737 group were 2.5, 5 and 10 μg/ml; the concentrations of ABT-737 of ABT-737 or DM-NCTD + ABT-737 group were 1.25, 2.5 and 5 μg/ml. The concentrations of nanoparticle groups were calculated according to the contents of DM-NCTD (2.5, 5 and 10 μg/ml). ^**^P < 0.01, ^*^P < 0.05 *vs* FA-LB(ABT-737)-(DM-NCTD@CHMSN) group; ^##^*P* < 0.01, ^#^*P* < 0.05 *vs* LB(ABT-737)-(DM-NCTD@CHMSN) group

The drug loading efficiency (DL%) of DM-NCTD loaded by CHMSN was 28.2%. In FA-LB(ABT-737)-(DM-NCTD@CHMSN), the encapsulation efficiency (EE%) of DM-NCTD and ABT-737 were 60.1 and 71.4%, while DL% of DM-NCTD and ABT-737 were 4.23 and 2.46%. DL% was decreased due to the increased weight after the coverage of LB. Based on the DL%, the content ratio of two drugs in the nanoparticles was beneficial to enhance the anti-tumor effect [5, 6]. The in vitro release of DM-NCTD and ABT-737 demonstrated that FA-LB(ABT-737)-(DM-NCTD@CHMSN) released the drugs in a sustained manner within 48 h, which could help drugs achieve a sustained antitumor effect (Additional file 2: Figure S1). H22 cells were incubated with free drugs and nanoparticle samples for 24 h to analyze the cytotoxicity in vitro, as depicted in Fig. 3a. The DM-NCTD + ABT-737 group exhibited the greater antitumor effect than DM-NCTD group and ABT-737 group, which result was consistent with the previous reports [5, 6]. FA-LB(ABT-737)-(DM-NCTD@CHMSN) group was better than LB(ABT-737)-(DM-NCTD@CHMSN) group, due to FR-mediated endocytosis mechanism [11]. Moreover, DM-NCTD + ABT-737 group (green column) exhibited the greater antitumor effect of than LB(ABT-737)-(DM-NCTD@CHMSN) group (purple column), and the FA-modified group (blue column) was better than the unmodified group and close to DM-NCTD + ABT-737 group (not significantly) in the 5 and 10 μg/ml of DM-NCTD. We believe that the cellular internalization mechanism of the free drug and drug-loaded nanoparticles contributed to this phenomenon. For DM-NCTD + ABT-737 group, the low molecular weight (186.16 Da) of DM-NCTD and the low molecular weight (813.43 Da) of ABT-737 might assist in their internalization into cells by direct diffusion. For the nanoparticles, the two drugs were released slowly in the influence of lipid bilayer and CHMSN, thus explaining why the free drug accumulated more quickly than LB(ABT-737)-(DM-NCTD@CHMSN) in H22 cells with 5 and 10 μg/ml of DM-NCTD. By comparison, the inhibition rate of the FA modified nanoparticle group enhanced significantly than unmodified nanoparticle group, due to FR-mediated endocytosis mechanism, which inhibition rate was close to that of DM-NCTD + ABT-737 group [11]. The results and in vitro release study showed the FA-LB(ABT-737)-(DM-NCTD@CHMSN) has the characteristics of the apparent anti-tumor efficacy and sustained drug release, which could provide reference for anti-tumor study in vivo.

Furthermore, we used Annexin V-FITC/PI to confirm the apoptotic effect. In Fig. 3b, d, we could found that FA-LB(ABT-737)-(DM-NCTD@CHMSN) group improved the two drug combination activity by enhancing the apoptosis region in H22 cells for 24 h. Besides, the mitochondrial membrane potential (MMP) is an important parameter for the function of mitochondria, which was closely related to apoptosis [23]. The influence of different groups on the MMP was measured by JC-1 detection. In Fig. 3c, e, FA-LB(ABT-737)-(DM-NCTD@CHMSN) resulted in a significant increase of JC-1 monomers compared with the other groups.

**Fig. 4:**
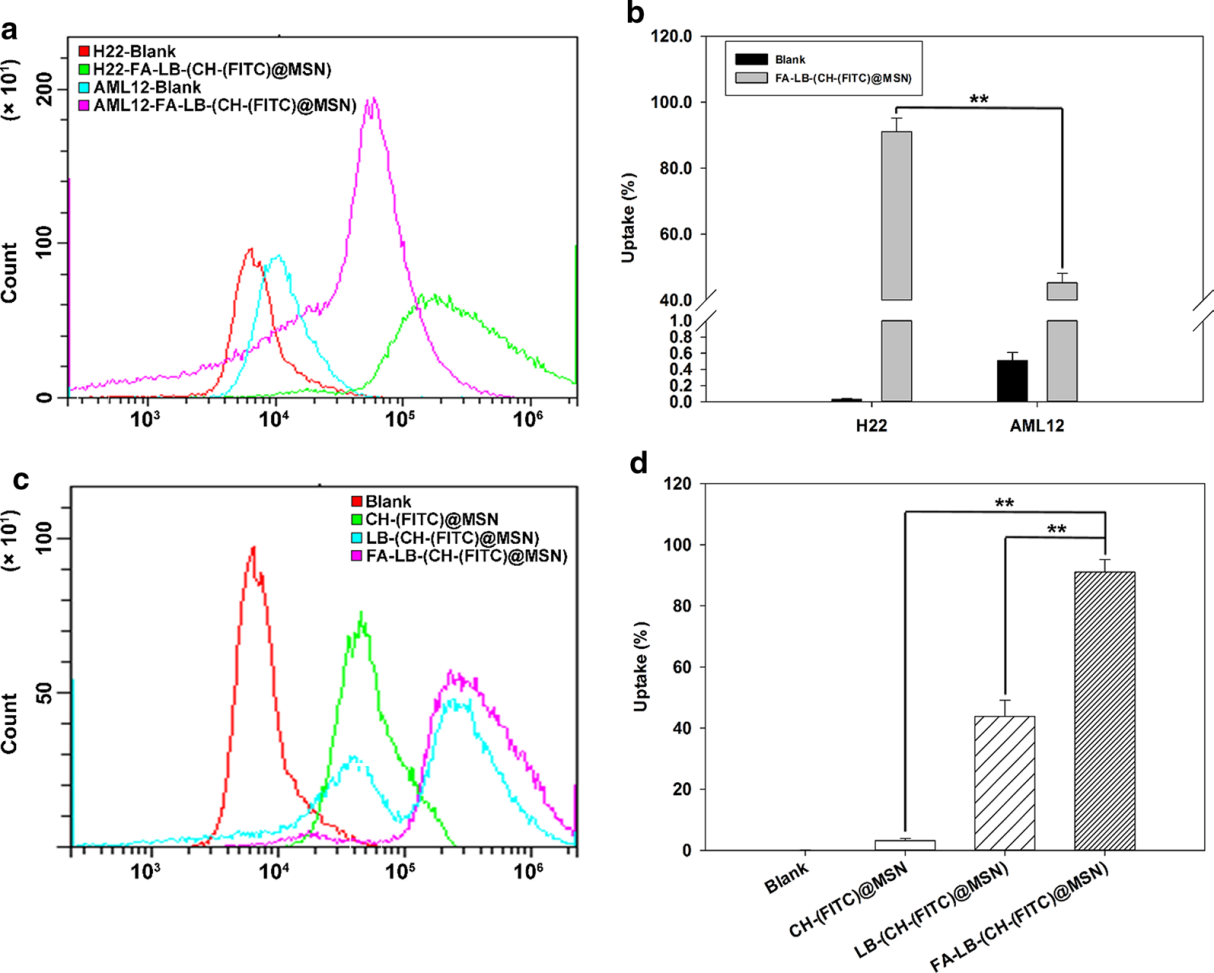
**a** Effect of cellular uptake of FA-LB-(CH-(FITC)@MSN) on both H22 and AML12 cells, respectively. **b** The quantitative analysis of percentage of cellular uptake of FA-LB-(CH-(FITC)@MSN) on H22 cells in comparison to that on AML12 cells (n = 3), ^**^P < 0.01: FA-LB-(CH-(FITC)@MSN) on H22 cells group *vs* FA-LB-(CH-(FITC)@MSN) on AML12 cells group. **c** Effect of cellular uptake of CH-(FITC)@MSN, LB-(CH-(FITC)@MSN) and FA-LB-(CH-(FITC)@MSN) on H22 cells, respectively. **d** The quantitative analysis of percentage of cellular uptake of CH-(FITC)@MSN, LB-(CH-(FITC)@MSN) and FA-LB-(CH-(FITC)@MSN) on H22 cells (n = 3), ^**^P < 0.01: CH-(FITC)@MSN and LB-(CH-(FITC)@MSN) *vs* FA-LB-(CH-(FITC)@MSN) group

The intracellular uptake of FITC-loaded nanoparticles was studied in H22 and AML12 cells using flow cytometry, which was used to quantify the fluorescent intensity of nanoparticle uptake by cells (Fig. 4). From Fig. 4a, b, the percentage of uptake cells treated with FA-LB-(CH-(FITC)@MSN) was significantly higher than that of AML12 cells treated with the same nanoparticles, which revealed that FA-LB-CHMSN may have the ability for a preferential targeting to the tumor cells, due to FR-mediated endocytosis.[11] The results also suggested that the carrier could have little effect on normal cells, which laid a foundation for further safety evaluation in vivo. From Fig. 4c, d, the fluorescent intensity and the percentage of uptake of the positive H22 cell population in FA-LB-(CH-(FITC)@MSN) group increased significantly, compared to CH-(FITC)@MSN or LB-(CH-(FITC)@MSN) group. The study indicated that FA-LB-CHMSN could enter H22 cells efficiently. Based on the results mentioned above, the in vivo anti-tumor study and preliminary toxicity evaluation were investigated.

**Fig. 5:**
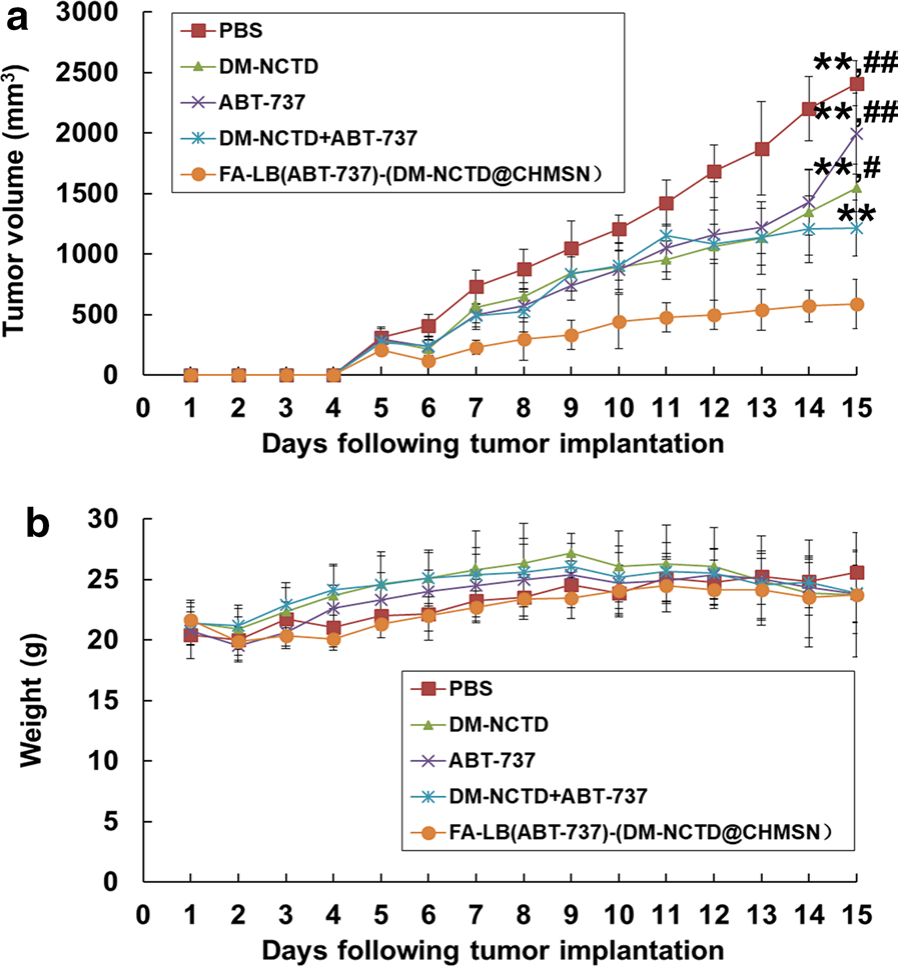
**a** Tumor growth in H22 tumor-bearing mice treated with free drugs and nanoparticle samples after H22 cells were injected at day 0 (administered on days 1–14, and killed on day 15; n = 6). **b** H22 tumor-bearing mice weight change of free drugs and nanoparticle samples after H22 cells were injected at day 0 (administered on days 1–14, and killed on day 15; n = 6). Notes: In free drug groups, DM-NCTD, 2 mg/kg (*i.v.*); ABT-737, 50 mg/kg (*i.p.*); FA-LB(ABT-737)-(DM-NCTD@CHMSN) group dosage was calculated according to the contents of DM-NCTD (2 mg/kg, *i.v.*). ^**^P < 0.01 *vs* FA-LB(ABT-737)-(DM-NCTD@CHMSN) group; ^##^*P* < 0.01, ^#^*P* < 0.05 *vs* DM-NCTD + ABT-737 group

**Fig. 6:**
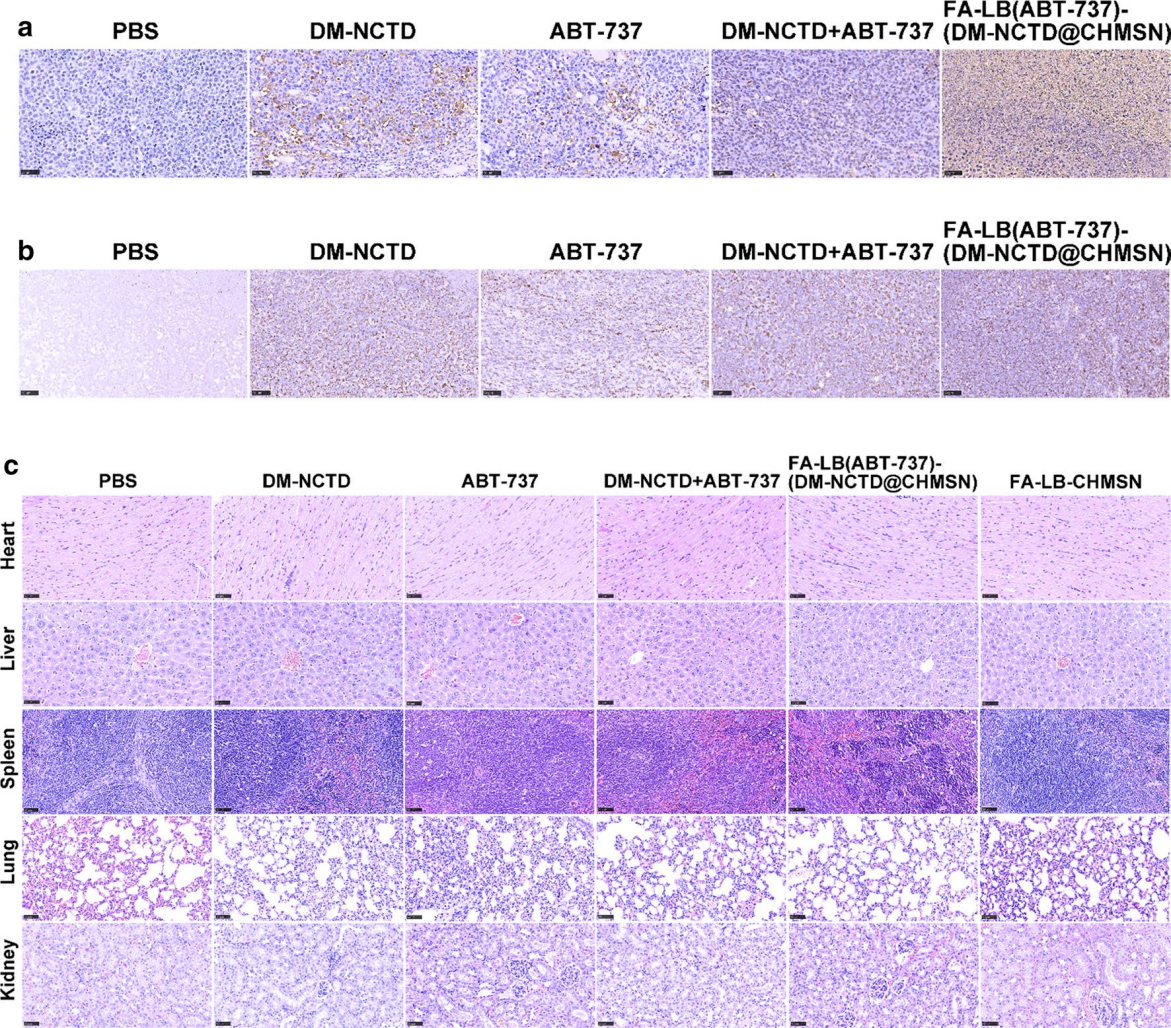
**a** Tumors stained with TUNEL (bar = 50 μm). **b** Cytochrome C expression was detected in tumor samples of the five experimental groups via immunohistochemistry (bar = 50 μm). **c** Tissues of H22 tumor-bearing mice stained with H&E after in vivo antineoplastic activity study (bar = 50 μm)

From Fig. 5a, DM-NCTD + ABT-737 group could enhance tumor inhibition in H22 tumor-bearing mice, in comparison with either DM-NCTD or ABT-737 group, which results revealed the effect of two drug combination therapies. However, both DM-NCTD and ABT-737 were eliminated rapidly in vivo, which limited their application.[7, 8] By comparison, we found that FA-modified nanoparticles showed the strongest tumor inhibition among those groups. The inhibition rate on tumor weight (IR_w_) was also determined (Additional file 3: Table S1). FA-LB(ABT-737)-(DM-NCTD@CHMSN) exhibited excellent antitumor activity, which was considerably enhanced compared with DM-NCTD + ABT-737 group. We are highly convinced that the synergistic co-delivery of DM-NCTD/ABT-737 loaded by FA-LB-CHMSN effectively improves tumor inhibition in the H22 tumor-bearing model. Figure 5b demonstrates a similar weight-change range of the H22 tumor-bearing mice during the experiment. Therefore, none of the treatments influenced the weight of the model mice. Tumor-cell apoptosis and the preliminary tissue toxicity were evaluated by TUNEL assays and H&E staining, and Cytochrome C expression was detected in tumor samples of the five experimental groups via immunohistochemistry, which was an important marker of cell apoptosis after mitochondrial damage [24]. In Fig. 6, FA-LB(ABT-737)-(DM-NCTD@CHMSN) induced more significant tumor-cell apoptosis and more Cytochrome C expression was detected, compared with the other groups (Fig. 6a, b), and none of the groups showed obvious toxicity to the tissues (Fig. 6c). Furthermore, there is no obvious toxicity of FA-LB-CHMSN to the tissues (Fig. 6c). Besides, the pharmacokinetics characteristics of FA-LB(ABT-737)-(DM-NCTD@CHMSN) and its antitumor mechanism action must be evaluated in further studies. The results could reveal whether DM-NCTD exists the characteristics of long-circulating in vivo by the influence of the nanoparticle and the findings of antitumor mechanism study are expected to lay the foundation for providing potential applications of FA-LB-CHMSN in anticancer treatment based on synergistic therapy.

## Conclusions

In conclusion, this research demonstrated that FA-LB(ABT-737)-(DM-NCTD@CHMSN) could be a potential nanocarrier for HCC treatment, which also provide a reference for the synergistic therapy of DM-NCTD and ABT-737.

## Supplementary information

**Additional file 1.** Supplementary Materials.

**Additional file 2: Figure S1.** In vitro release profiles of the two drugs from FA-LB(ABT-737)-(DM-NCTD@CHMSN) within 48 hours in PBS (pH 7.4) containing 0.1% of Tween 80 (v/v) (n = 3).

**Additional file 3: Table S1.** Tumor weight and IRw in H22 tumor-bearing mice after H22 cells injected at day 0 (administered on days 1–14, killed and measured on day 15, n = 6)

## Data Availability

The datasets used and/or analysed during the current study are available from the corresponding author on reasonable request.
