# Supplementary material for: Synergistic co-delivery of diacid metabolite of norcantharidin and ABT-737 based on folate-modified lipid bilayer-coated mesoporous silica nanoparticle against hepatic carcinoma
Source: J Nanobiotechnology. 2020 Aug 18;18:114. doi: 10.1186/s12951-020-00677-4 (PMC7437073; doi:10.1186/s12951-020-00677-4)
Supplement: Supplementary file 1 — Additional file 1. Supplementary Materials. [file 12951_2020_677_MOESM1_ESM.docx]

**2. Method**

2.1 Materials

NCTD (purity > 98%) was purchased from J&K Scientific (Beijing, PRC). DM-NCTD was converted from NCTD (1.11:1, molar ratio). ABT-737 was purchased from MedChemExpress (USA), while 1,2-distearoyl-sn-glycero-3-phosphatidylcholine (DSPC), 1,2-distearoyl-sn-glycero-3-phosphoethanolamine-N-[methoxy(PEG)-2000] (DSPE-PEG2000), DSPE-PEG2000-FA and cholesterol were all purchased from Resenbio (Xi’an, PRC). Tetraethylorthosilicate (TEOS), cetyltrimethylammonium bromide (CTAB), (3-aminopropy) trimethoxysilane (APTMS), chlorodimethyloctadecylsilane (CH) and fluorescein isothiocyanate (FITC) were obtained from Sigma-Aldrich (St. Louis, USA). Roswell Park Memorial Institute (RPMI) 1640 medium, Dulbecco’s modified Eagle’s medium (DMEM), DMEM/F12 (Shanghai Cell Center of the Chinese Academy of Medical Science), Fetal bovine serum (FBS), penicillin, streptomycin were purchased from Gibco BRL (Gaithersberg, MD, USA). [3-(4,5-dimethylthiazol-2-yl)-2,5-diphenyltetrazolium bromide] (MTT) was purchased from Sigma-Aldrich (St. Louis, MO). Annexin V-FITC/PI Apoptosis Detection Kit and Flow Cytometry Mitochondrial Membrane Potential Detection Kit were both obtained from BD Bioscience (USA). Cytochrome c (D18C7) Rabbit mAb for immunohistochemistry was obtained from Cell Signaling Technology (USA). Other chemicals and solvents were all of analytical grade.

2.2 Cell culture and animals

The murine hepatic AML12 cell line was obtained from the Shanghai Cell Center of the Chinese Academy of Medical Science (Shanghai, PRC). The murine hepatic AML12 cell were cultured in DMEM/F12 medium supplemented with 10% FBS. The H22 murine hepatoma cell line was obtained from Chinese Academy of Medical Sciences (Beijing, PRC) and were cultured in RPMI 1640 medium supplemented with 10% FBS. The two cells were both cultured at 37°C in a 5% CO_2_ atmosphere, containing 100 U/ml of penicillin and 100 µg/ml of streptomycin.

Male and female Kunming mice (20–22 g) were kept by the Experimental Animal Research Center, Zhejiang Chinese Medical University (Hangzhou, PRC). All animal procedures were performed in accordance with Health Guidelines for the Care and Use of Laboratory Animals of Zhejiang Chinese Medical University, and the experiments were approved by the Animal Ethics Committee of Zhejiang Chinese Medical University.

2.3 Preparation of Amino-modified MSN

Amino-modified MSN were prepared as described in the previous report with minor modifications.[[1](#_ENREF_1)] The total process contained three following steps. First of all, CTAB and NaOH (3:1, w/w) were dissolved into deionized water and stirred at 80 °C. TEOS was added within 20 min under stirring for 4 h. Secondly, the mixture was centrifuged (10,000 rpm, 10 min). And then, the material was washed ethanol and diluted hydrochloric acid. At last, the surfaces of products were modified with APTMS, which was denoted as amino-modified MSN. And then, the mixture was centrifuged and washed with ethanol and water, and was dispersed in PBS prior to use.

2.4 Preparation of dual drug-loaded FA-LB-CHMSN

CH was added to CHCl_3_ (1:100, v/v) and amino-modified MSN under stirring for overnight, which were washed with hexane and dried in air.[[2](#_ENREF_2)] 5 mg CHMSN was dispersed in 5 ml DM-NCTD solution (0.5 mg/ml) and stirred overnight. The obtained DM-NCTD@CHMSN was collected by centrifugation. And then, FA-LB(AT-737)-(DM-NCTD@CHMSN) were prepared by modified thin film hydration method as previously described.[[3](#_ENREF_3)] In brief, DSPC, DSPE-PEG2000, DSPE-PEG2000-FA and cholesterol (2:0.11:0.017:1 molar ratio) were dissolved in chloroform. DM-NCTD@CHMSN was added with a weight ratio of 20% to the lipids. The process was followed by evaporating under negative pressure to form a lipid layer surrounding the particles using a rotary evaporator (BUCHI, SWITZERLAND) at 60 °C. Then, at a molar ratio of 1:10 to DM-NCTD, ABT-737 was added to the lipid mixture before the thin-film was hydrated.[[4](#_ENREF_4)] The supernatant were removed by centrifugation at 10,000 rpm for 5 min for the isolation. The nanoparticle denoted as FA-LB(AT-737)-(DM-NCTD@CHMSN) were subsequently washed with PBS and dispersed in PBS prior to use. LB(AT-737)-(DM-NCTD@CHMSN) were prepared by the similar method as mentioned above without DSPE-PEG2000-FA. The amount of DM-NCTD or ABT-737 was determined by high-performance liquid chromatography (HPLC, Agilent 1200, USA). The encapsulation efficiency (EE%) and drug loading efficiency (DL%) were estimated as follows:

$$\text{EE\% = }\frac{\text{W}_{\text{drug in nanoparticle}}\text{ }}{\text{W}_{\text{total drug}}}\text{ ×100 DL\% = }\frac{\text{W}_{\text{drug in nanoparticle}}}{\text{W}_{\text{nanoparticle}}}\text{ ×100}$$

A Zorbax SB-C18 column (4.6 × 250 mm, 5 μm) was employed. The mobile phase for DM-NCTD was mentioned as the previously study.[[5](#_ENREF_5)] The mobile phase for ABT-737 was 0.1% H_3_PO_4_ in 100% acetonitrile and 0.1% H_3_PO_4_ in 100% water, which was used for was used for gradient elution at 0.5 ml/min. The wavelength of the ultraviolet detector was 300 nm.

2.5 Preparation of FITC@MSN and CH-(FITC)@MSN

FITC was applied to observe the influence of CH before and after CHMSN was prepared. MSN were dispersed and incubated in dark with FITC (5:1, w/w, 25 °C) for 12 h under stirring. The FITC@MSN was collected by centrifugation. And then, CH-(FITC)@MSN was prepared as 2.4 mentioned about the preparation of CHMSN. At last, the dispersion of FITC@MSN and CH-(FITC)@MSN were observed in water/chloroform mixed solvent. Furthermore, FA-LB-(CH-(FITC)@MSN) and LB-(CH-(FITC)@MSN) were prepared by the similar method as mentioned in 2.4, which were applied in cellular uptake study.

2.6 Characterization

Transmission electron microscopy (TEM, JEM 2010, JEOL) of MSN and CHMSN and Cryo-TEM (Tecnai G2 F20) of LB-CHMSN and FA-LB-CHMSN were obtained. The surface analysis was performed by nitrogen sorption isotherms at 77 K (Quantachrome, USA). Pore size distribution and surface area were evaluated by Barrett Joyner Halenda (BJH) method and Brunauer Emmett Teller (BET) method. Moreover, Powder X-ray patterns (XRD) were recorded (PANalytical, Holland). The particles and zeta potentials of the samples were measured by Zetasizer 3000HS (Malvern Instruments, Malvern, UK). The infrared feature of CH from CHMSN were measured by FT-IR spectrophotometer (Thermo Fisher Scientific, USA).

2.7 *In vitro* release study

DM-NCTD and ABT-737 release from the FA-LB(ABT-737)-(DM-NCTD@CHMSN) was measured by dialysis (2,000 Da cutoff). During the experiment, the samples were placed in PBS (pH 7.4) containing 0.1% of Tween 80 (v/v), and stirred for 48 hours at 37 °C. Aliquots were withdrawn from the beaker and replaced with equal volumes of the medium at different time points. The DM-NCTD or ABT-737 concentrations were measured by HPLC.

2.8 *In vitro* cytotoxicity

The *in vitro* antineoplastic activity of nanoparticles loaded with DM-NCTD/ABT-737 was determined by the cytotoxicity to H22 cells. Cells in logarithmic growth were seeded into 96-well plates at a density of 10^4^ cells per well for 24 hours, and then the medium was exchanged for fresh medium. The cells were then treated with 100 μl of sterile samples, including DM-NCTD, ABT-737, DM-NCTD+ABT-737, LB(ABT-737)-(DM-NCTD@CHMSN) and FA-LB(ABT-737)-(DM-NCTD@CHMSN) groups. Concentrations of DM-NCTD in DM-NCTD group or DM-NCTD+ABT-737 group were 2.5, 5 and 10 μg/ml, while concentrations of ABT-737 in ABT-737 group or DM-NCTD+ABT-737 group were 1.25, 2.5 and 5 μg/ml. Moreover, the concentrations of nanoparticle groups were calculated according to the contents of DM-NCTD (2.5, 5 and 10 μg/ml). Next, the cells were incubated for 24 hours. And then, 20 μl of a 5 mg/ml MTT solution in PBS was added, and the plate was incubated for 4 hours. The supernatant was then removed, and 100 μl of DMSO was added to each well. After the formazan crystals had dissolved, the absorbance of the resulting solution was measured at 570 nm using a model 680 Microplate Reader (Bio-Rad, USA).

2.9 Apoptosis analysis

Apoptosis in cells treated with samples was examined by flow cytometry (BD Accuri C6) using Annexin V-FITC/PI Apoptosis Detection Kit according to the manufacturer's instructions.

2.10 The mitochondrial activity

The mitochondrial activity was examined by Flow Cytometry Mitochondrial Membrane Potential Detection Kit according to the manufacturer's instructions.

2.11 Cellular uptake

In cellular uptake study, the concentrations of FITC of CH-(FITC)@MSN, LB-(CH-(FITC)@MSN) or FA-LB-(CH-(FITC)@MSN) were 5 μg/ml. H22 murine hepatoma cells and the murine hepatic AML12 cells were seeded at a cell density of 2 × 10^5^ cells per well in 6-well plates and incubated for 24 hours. And then, the culture medium was removed. The distinct expression pattern of FR in normal and malignant tissues makes FA-modified nanoparticles an ideal target for drug delivery. FA specifically promotes cancer-cell uptake through FR-mediated endocytosis.[[6](#_ENREF_6)] To test the FA-LB-CHMSN’s ability of specific targeting to the tumor cells by the influence of FA, FA-LB-(CH-(FITC)@MSN) were then added in H22 cells and AML12 cells, respectively. Besides, to investigate cell uptake effect of different nanoparticles in tumor cells, CH-(FITC)@MSN, LB-(CH-(FITC)@MSN) and FA-LB-(CH-(FITC)@MSN) were then added in H22 cells. For 48 hours, the cells were washed with PBS three times, and resuspended in 1 ml of PBS for flow cytometric analysis.

2.12 *In vivo* anti-tumor study and preliminary toxicity evaluation

The preparation of the H22 tumor-bearing mouse model was performed as the previously study.[[5](#_ENREF_5)] And then, the model mice were randomly assigned to the following experimental groups: PBS, DM-NCTD, ABT-737, DM-NCTD+ABT-737 and FA-LB(ABT-737)-(DM-NCTD@CHMSN). DM-NCTD in DM-NCTD group or DM-NCTD+ABT-737 group was injected with 2 mg/kg through the tail vein, while ABT-737 in ABT-737 group or DM-NCTD+ABT-737 group was intraperitoneally administered 50 mg/kg.[[7](#_ENREF_7), [8](#_ENREF_8)] Moreover, the FA-LB(ABT-737)-(DM-NCTD@CHMSN) group was injected through the tail vein and its dosage was calculated according to the content of DM-NCTD (2 mg/kg). Besides, to investigate the preliminary toxicity of the empty nanoparticles, the model mice were randomly assigned to the FA-LB-CHMSN group, which dosage was calculated as FA-LB(ABT-737)-(DM-NCTD@CHMSN) group dosage

Tumor growth was investigated in H22 tumor-bearing mice administrated with five different experimental groups on days 1–14 after H22 cells were injected on day 0 (n = 6). The mice were also weighed every day. The mice were sacrificed on day 15, and then subcutaneous tumors were carefully collected and weighed, while the tumor size was measured every day on days 1–15. The inhibition rate on tumor weight (IR_w_) and the tumor volumes (V) were calculated as follows:

$$\text{IR}_{\text{W}}\text{\% = [1}-\frac{\text{W}_{\text{drug}}}{\text{W}_{\text{control}}}\text{] ×100 V= }\frac{\text{Length × }\text{Width}^{\text{2}}}{\text{2}}$$

At last, the major organs (heart, liver, spleen, lung, and kidney) and tumors were collected and fixed in a 4% formaldehyde solution for 24 hours at room temperature. Tumor-cell apoptosis was detected by TUNEL assay, while the tissues were stained with H&E, and Cytochrome C expression was detected in tumor samples of the five experimental groups via immunohistochemistry, according to the manufacturer’s instructions. Photographs were captured under a microscope (Nikon eclipse 80i, Japan).

2.13 Statistical analysis

Data are expressed as mean ± standard deviation. *P* < 0.05 was considered statistically significant, and extreme significance was set at *P* < 0.01.

**References**

1. Wu X, Wang Z, Zhu D, Zong S, Yang L, Zhong Y, Cui Y: **pH and thermo dual-stimuli-responsive drug carrier based on mesoporous silica nanoparticles encapsulated in a copolymer-lipid bilayer.** *ACS Appl Mater Interfaces* 2013, **5:**10895-10903.

2. Fei W, Zhang Y, Han S, Tao J, Zheng H, Wei Y, Zhu J, Li F, Wang X: **RGD conjugated liposome-hollow silica hybrid nanovehicles for targeted and controlled delivery of arsenic trioxide against hepatic carcinoma.** *Int J Pharm* 2017, **519:**250-262.

3. Ren J, Shen S, Wang D, Xi Z, Guo L, Pang Z, Qian Y, Sun X, Jiang X: **The targeted delivery of anticancer drugs to brain glioma by PEGylated oxidized multi-walled carbon nanotubes modified with angiopep-2.** *Biomaterials* 2012, **33:**3324-3333.

4. Choi JY, Ramasamy T, Kim SY, Kim J, Ku SK, Youn YS, Kim JR, Jeong JH, Choi HG, Yong CS, Kim JO: **PEGylated lipid bilayer-supported mesoporous silica nanoparticle composite for synergistic co-delivery of axitinib and celastrol in multi-targeted cancer therapy.** *Acta Biomater* 2016, **39:**94-105.

5. Liu MC, Liu L, Wang XR, Shuai WP, Hu Y, Han M, Gao JQ: **Folate receptor-targeted liposomes loaded with a diacid metabolite of norcantharidin enhance antitumor potency for H22 hepatocellular carcinoma both in vitro and in vivo.** *Int J Nanomedicine* 2016, **11:**1395-1412.

6. Antony AC: **The biological chemistry of folate receptors.** *Blood* 1992, **79:**2807-2820.

7. Hikita H, Takehara T, Shimizu S, Kodama T, Shigekawa M, Iwase K, Hosui A, Miyagi T, Tatsumi T, Ishida H, et al: **The Bcl-xL inhibitor, ABT-737, efficiently induces apoptosis and suppresses growth of hepatoma cells in combination with sorafenib.** *Hepatology* 2010, **52:**1310-1321.

8. Wang Q, Zhang L, Hu W, Hu ZH, Bei YY, Xu JY, Wang WJ, Zhang XN, Zhang Q: **Norcantharidin-associated galactosylated chitosan nanoparticles for hepatocyte-targeted delivery.** *Nanomedicine* 2010, **6:**371-381.
