# Supplementary material for: Synergistic co-delivery of diacid metabolite of norcantharidin and ABT-737 based on folate-modified lipid bilayer-coated mesoporous silica nanoparticle against hepatic carcinoma
Source: J Nanobiotechnology. 2020 Aug 18;18:114. doi: 10.1186/s12951-020-00677-4 (PMC7437073; doi:10.1186/s12951-020-00677-4)
Supplement: Supplementary file 2 — Additional file 2: Figure S1. In vitro release profiles of the two drugs from FA-LB(ABT-737)-(DM-NCTD@CHMSN) within 48 hours in PBS (pH 7.4) containing 0.1% of Tween 80 (v/v) (n = 3). [file 12951_2020_677_MOESM2_ESM.docx]

**
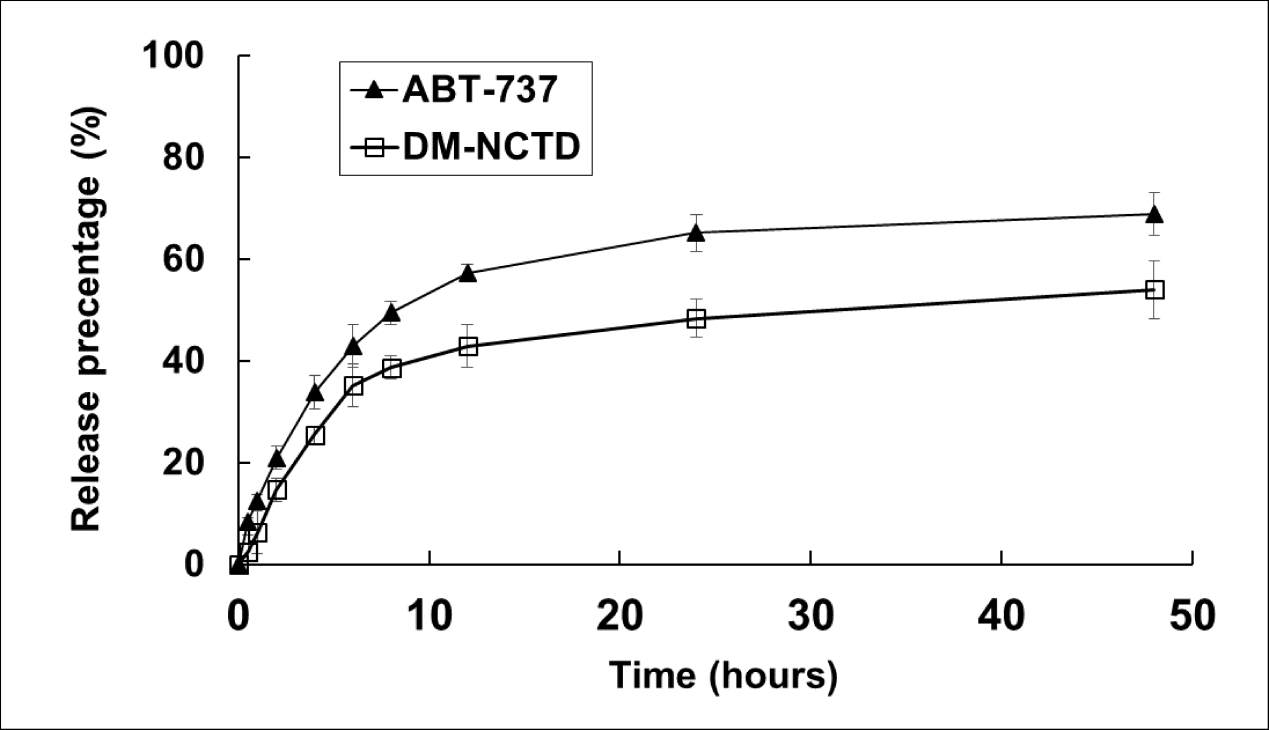
**

**Fig. S1** *In vitro* release profiles of the two drugs from FA-LB(ABT-737)-(DM-NCTD@CHMSN) within 48 hours in PBS (pH 7.4) containing 0.1% of Tween 80 (v/v) (n = 3).
