# Supplementary material for: Synergistic co-delivery of diacid metabolite of norcantharidin and ABT-737 based on folate-modified lipid bilayer-coated mesoporous silica nanoparticle against hepatic carcinoma
Source: J Nanobiotechnology. 2020 Aug 18;18:114. doi: 10.1186/s12951-020-00677-4 (PMC7437073; doi:10.1186/s12951-020-00677-4)
Supplement: Supplementary file 3 — Additional file 3: Table S1. Tumor weight and IRw in H22 tumor-bearing mice after H22 cells injected at day 0 (administered on days 1–14, killed and measured on day 15, n = 6) [file 12951_2020_677_MOESM3_ESM.docx]

**Table S1** Tumor weight and IR_w_ in H22 tumor-bearing mice after H22 cells injected at day 0 (administered on days 1–14, killed and measured on day 15, n = 6)

| **Group** | **Tumor weight (g)** | **IR_w_ (%)** |
| --- | --- | --- |
| PBS | 2.86 ± 0.21**^,##^ | - |
| DM-NCTD | 1.87 ± 0.28*^,#^ | 34.6 |
| ABT-737 | 2.26 ± 0.15**^,##^ | 21.0 |
| DM-NCTD+ABT-737 | 1.55 ± 0.13* | 45.8 |
| FA-Lipo(ABT-737)-CHMSN@DM-NCTD | 0.87 ± 0.14 | 69.6 |

Notes: ***P* < 0.01, **P* < 0.05 *vs* FA-LB(ABT-737)-(DM-NCTD@CHMSN) group; ^##^*P* < 0.01, ^#^*P* < 0.05 *vs* DM-NCTD+ABT-737 group. Data presented as mean ± standard deviation.
